# Task-Driven Learned Hyperspectral Data Reduction Using End-to-End Supervised Deep Learning

**DOI:** 10.3390/jimaging6120132

**Published:** 2020-12-02

**Authors:** Mathé T. Zeegers, Daniël M. Pelt, Tristan van Leeuwen, Robert van Liere, Kees Joost Batenburg

**Affiliations:** 1 Centrum Wiskunde & Informatica, Science Park 123, 1098 XG Amsterdam, The Netherlands; d.m.pelt@cwi.nl (D.M.P.); t.van.leeuwen@cwi.nl (T.v.L.); robert.van.liere@cwi.nl (R.v.L.); joost.batenburg@cwi.nl (K.J.B.); 2 Leiden Institute of Advanced Computer Science, Niels Bohrweg 1, 2333 CA Leiden, The Netherlands; 3 Mathematical Institute, Utrecht University, Budapestlaan 6, 3584 CD Utrecht, The Netherlands; 4 Faculteit Wiskunde en Informatica, Technical University Eindhoven, Groene Loper 5, 5612 AZ Eindhoven, The Netherlands

**Keywords:** hyperspectral imaging, feature extraction, compression, machine learning, deep learning, convolutional neural network, segmentation

## Abstract

An important challenge in hyperspectral imaging tasks is to cope with the large number of spectral bins. Common spectral data reduction methods do not take prior knowledge about the task into account. Consequently, sparsely occurring features that may be essential for the imaging task may not be preserved in the data reduction step. Convolutional neural network (CNN) approaches are capable of learning the specific features relevant to the particular imaging task, but applying them directly to the spectral input data is constrained by the computational efficiency. We propose a novel supervised deep learning approach for combining data reduction and image analysis in an end-to-end architecture. In our approach, the neural network component that performs the reduction is trained such that image features most relevant for the task are preserved in the reduction step. Results for two convolutional neural network architectures and two types of generated datasets show that the proposed Data Reduction CNN (DRCNN) approach can produce more accurate results than existing popular data reduction methods, and can be used in a wide range of problem settings. The integration of knowledge about the task allows for more image compression and higher accuracies compared to standard data reduction methods.

## 1. Introduction

In hyperspectral imaging, data are collected for a large number of spectral bins from a wavelength range in the electromagnetic spectrum. It is used in various fields [1], including agriculture classification [2,3], medical imaging [2,4,5], luggage and cargo inspection [2,6,7] and food quality assessment [8], as well as with energy-dispersive X-ray spectroscopy (EDX) and electron energy loss spectroscopy (EELS) [9]. In addition to the spatial dimensions, hyperspectral data include the spectral dimension which is typically large [10], often in the order of $10^{2}$ to $10^{3}$ spectral bins [11]. The data have rich information for image processing tasks (for instance segmentation and classification) [11]. Hyperspectral imaging can circumvent calibration issues found in (multi)spectral imaging with a low number of energy bins, such as carefully setting spectral measurement ranges.

A key challenge for hyperspectral imaging systems is handling the size of the data, which can be prohibitively large for online processing [12]. Efficient data compression is essential to save storage and reduce transmission load [13], for instance in remote sensing with satellites sending spectral images to the Earth or in high-throughput food quality assessment tasks [1,8]. For industrial applications, training and running algorithms for classification tasks on full hyperspectral data may be very time consuming [14]. Therefore, data reduction steps need to be carried out to reduce data redundancy and size. However, it is not known a priori which spectral bins contain important information, and combining information from many bins may be required for the data interpretation. In addition, bins may contain a low signal-to-noise ratio [9,15], possibly exacerbated by limited acquisition times in some applications. More, bins that are located close to each other are highly correlated which results in redundant information [16].

The goal of this paper is to propose a new convolutional neural network (CNN)-based approach for hyperspectral data reduction that combines high computational efficiency with strong data reduction (down to just 1 or 2 channels), by making effective use of the relation between the spectral signatures in the data and the specific task that needs to be performed. By attaching a data reduction network to a CNN component for segmentation, the combined network simultaneously learns how to effectively reduce the input data to a low number of images, eliminate spectral redundancy and successfully perform a given task, without the need for parameter tuning. The network adapts to different problem settings and learns how to effectively compress the data for the problem while maintaining accurate segmentation with fast processing times. We assess the performance of the method on a simulated dataset consisting of attenuation-based hyperspectral X-ray projection images, as well as on a simulated dataset based on spectral properties found in remote sensing. These multi-image datasets allow for the method to be evaluated without risks of information leakage between training and test sets [17]. We show that the method is applicable to different CNN architectures by applying it on a Mixed-Scale Dense (MSD) [18] and a U-Net architecture [19]. The results show that our method is robust to noise and to cases where many different materials or classes are involved, for which standard data reduction methods, such as Principle Component Analysis (PCA) and Linear Discriminant Analysis (LDA), are not sufficient. To summarize, the main contribution of this paper is providing a flexible learned supervised data reduction approach with convolutional neural networks with spectral data reduction to a very limited number of images, while retaining high segmentation accuracies.

The remainder of this paper is structured as follows. Section 2 gives an overview of methods for spectral data reduction. In Section 3, we introduce notation and the general set of functions in convolutional neural networks to optimize. In addition, we give the problem statement for supervised segmentation and a brief explanation of the most widely used hyperspectral data reduction methods. Most importantly, we introduce our end-to-end hyperspectral data reduction method. In Section 4, we describe our experimental setup and the CNN architectures and datasets that are used, including a description of the creation of our simulated attenuation-based hyperspectral X-ray image dataset and simulated remote sensing dataset. Then, we outline the experiments and discuss the results of the data reduction approaches. Section 5 discusses the introduced method and the results, and additionally gives some further possibilities for future research. Section 6 summarizes the paper and presents the conclusions.

## 2. Related Work

As a result of the importance of data reduction in practical applications, a wide variety of approaches have been developed in earlier work. Two approaches for reducing the dimensionality of a hyperspectral image are hyperspectral band selection and feature extraction methods. Hyperspectral band selection methods select a small number of the bands (bins) to be used for the imaging task, based on searching, ranking, clustering or learning methods [12]. Hyperspectral feature extraction methods project the data into a new feature space with a lower dimension. While it changes the meaning of the data, more (combined) information can be stored in the lower dimensional images than the selected bins of band selection methods. In feature extraction methods, a wider range of reduced images can be found and used for the specific task to be carried out than in band selection methods. Common approaches for feature extraction include Principal Component Analysis (PCA) [20,21] and Linear Discriminant Analysis (LDA) [1,22,23,24], which are popular for their low complexity and absence of parameters. Other common data reduction techniques include Nonnegative Matrix Factorization (NMF) [25,26], Independent Component Analysis (ICA) [21,26,27,28] and many variants of PCA [21].

Popular feature extraction methods are fast and do not require parameter tweaking. However, since no task-specific information is used in commonly used unsupervised data reduction methods, features that are important for the given task (i.e., segmentation, classification) may not be preserved in the reduction step. Additionally, other problems such as the inability of PCA to deal with noisy channels and LDA generating only at most one reduction image less than the number of classes [23], as well as the linear nature of the these transformations make these approaches less suitable for complex data and feature distributions [29].

There is a wide range of other linear and non-linear data reduction approaches that requires different prior knowledge on the data for the image processing task [30]. For example, Kernel PCA (KPCA) makes the transformation of PCA non-linear, but requires the selection of a suitable kernel [31,32] and introduces the need for parameter tuning. In Locally Linear Embedding (LLE) and similar manifold learning methods, one or more parameters have to be chosen, and the optimal values are different for every dataset [33]. In several cases, the classical linear data reduction methods can outperform the non-linear data reduction techniques [34].

Convolutional neural networks (CNNs) are a powerful tool for classification and segmentation tasks [35,36,37]. These have the property to generalize well, as they can non-linearly extract distinctive spatial [38] and spectral properties [39] on different scales for segmentation tasks on noisy data. The current convolutional neural networks for hyperspectral imaging can be classified into three categories [21,38,40]. Spectral CNN methods apply one-dimensional convolutions in the spectral dimension to classify each pixel. These methods do not take into account that essential spectral information for classification may be located in very distant bins. Additionally, these methods also disregard spatial information [41]. Spatial CNN methods first reduce the data with a separate method, for example with PCA. The pixels in the remaining feature maps are then classified using 2D convolutions. This can, for example, be used when executing on-ground recovery of image information by a CNN after compression on-board of a satellite [42]. In these approaches, feature extraction and CNN classification are disconnected [2,43], so the feature extraction is not tailored to the CNN classification task. Spectral-spatial CNN methods take both spectral properties and spatial information into account and many possible designs and strategies can be developed for this, making this set of approaches highly flexible [40]. Data reduction can be integrated (both explicitly and implicitly) into the architecture of this type of CNN. Possibilities include performing 1D convolutions in the spectral dimension and in different layers before applying 2D convolutions in the spatial dimensions (1D + 2D CNNs) [44,45] or applying convolutions in all dimensions simultaneously (3D CNNs) [45]. However, with 3D CNNs, it is not possible to retrieve purely spectrally reduced images. Additionally, some of these spectral-spatial CNN approaches require hyperparameters to be properly tuned [39].

For classification methods that use spectral-spatial CNNs, the computation time can increase significantly with high-dimensional data [41,46]. In addition, the large number of training parameters makes the network difficult to train and easy to overfit [47], especially if only a few training examples are available, referring to the limited amount of labeled data that is available in hyperspectral imaging [48]. Therefore, some CNN-based approaches still need simple reduction methods such as PCA as a preprocessing step to keep computation times tractable [48,49], but a learned data reduction approach may outperform these standard reduction approaches [50]. Most current methods reduce to a relatively large number of reduction images (i.e., 32). In contrast, in this work we introduce a learned data reduction approach for CNNs to reduce data to a very limited number of channels (i.e., 1 or 2). By adding a data reduction network to the CNN and training the combined network in an end-to-end fashion, the data reduction becomes task-specific and can be applied with high compression, low computation times and without parameter tuning.

## 3. Materials and Methods

### 3.1. Notation and Concepts

#### 3.1.1. Hyperspectral Imaging

We consider the supervised hyperspectral image segmentation problem. A hyperspectral image is a three-dimensional image $x\in R^{N_{b}\times m\times n}$ with two spatial dimensions of size *m* and *n* and one spectral dimension of size $N_{b}$. The number of spectral bins $N_{b}$ is typically large compared to multispectral images, i.e., between about 100 to 1000 [11].

A segmented image is an image $y\in C^{m\times n}$ in which a class is assigned to each pixel from a finite set C of classes. A segmentation *y* of an image *x* divides the image into regions where the pixels have similar characteristics. For instance, it can divide a hyperspectral satellite image up into regions of classes including water, roads, vegetation, etc. We assume the existence of a true segmentation function $F_{s}:R^{N_{b}\times m\times n}\to C^{m\times n}$ that maps a hyperspectral image *x* to its segmented image $y=F_{s}\left(x\right)$. The problem, of course, is that this underlying function $F_{s}$ is generally not known. Therefore, the aim is to find an approximating segmentation function *F* such that $F\approx F_{s}$. Note that both spectral and spatial information are needed for a good segmentation (for example, vegetation can have the same shape but different spectral reflectance, and roads can have different shapes but similar spectral properties).

#### 3.1.2. Supervised Learning and Neural Networks

To solve the problem of finding an appropriate function *F*, supervised learning can be used. In this setting, a set of examples ${\left\{\left(x_{i},y_{i}\right)\right\}}_{i=1}^{N^{\mathrm{im}}}$ of hyperspectral images with their segmentations is available, with $N^{\mathrm{im}}$ being the number of images. The aim is to approximate the function $F_{s}$ based on segmentation training data with $y_{i}=F_{s}\left(x_{i}\right)$ for every *i*. In other words, the problem can be summarized as:

(1) $$\begin{array}{c} \mathrm{Find}\;a\;\mathrm{function}\;F:R^{N_{b}\times m\times n}\to C^{m\times n}\;\mathrm{such}\;\mathrm{that}\;F\left(x_{i}\right)\approx y_{i}\;\mathrm{for}\;\mathrm{every}\;i. \end{array}$$

The set of images and their segmentations can be partitioned into training, validation and test sets. To solve the supervised hyperspectral segmentation problem, the aim is to find a function $F:R^{N_{b}\times m\times n}\to C^{m\times n}$ such that the loss *L*, the error between the predicted classes by *F* from the training examples and their respective target images, is minimized:

(2) $$\begin{array}{c} \min_{F}\sum_{i=1}^{N^{\mathrm{train}}}L\left(F\left(x_{i}^{\mathrm{train}}\right),y_{i}^{\mathrm{train}}\right). \end{array}$$

To prevent overfitting on the training data, it is evaluated on a separate validation set ${\left\{\left(x_{i}^{\mathrm{val}},y_{i}^{\mathrm{val}}\right)\right\}}_{i=1}^{N^{\mathrm{val}}}$. The error on this set determines whether training should be continued or not by defining a stopping criterion. Subsequently, the function is tested on a separate test set ${\left\{\left(x_{i}^{\mathrm{test}},y_{i}^{\mathrm{test}}\right)\right\}}_{i=1}^{N^{\mathrm{test}}}$ to assess the overall performance.

**Figure 1:**
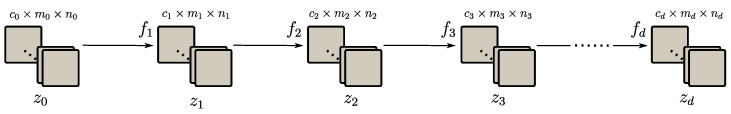
Schematic architecture of a neural network for image processing. Squares denote feature map channels and arrows denote function inputs. The depth of the network is given by *d*.

A common approach to find a suitable function *F* to satisfy Equation (1) with supervised learning is to parameterize it as a neural network. In many popular neural network architectures for imaging, the input is passed on from layer to layer to create feature maps, denoted by $z_{i}\in R^{c_{i}\times m_{i}\times n_{i}}$, where $c_{i}$ is the number of channels in the feature map of layer *i*. The structure is schematically shown in Figure 1, and we adopt notation from [18]. To finally produce the output feature map, the feature map $z_{0}\in R^{c_{0}\times m_{0}\times n_{0}}$ in the input layer is iteratively passed on from layer i−1 to *i* to produce feature maps $f\left(z_{i-1}\right)=z_{i}$ with $f_{i}:R^{c_{i-1}\times m_{i-1}\times n_{i-1}}\to R^{c_{i}\times m_{i}\times n_{i}}$. In networks for segmentation problems, the number of channels $c_{d}$ in the output layer is equal to the number of classes $N_{c}$, and the feature map $z_{d}$ contains probability maps for every class.

**Figure 2:**
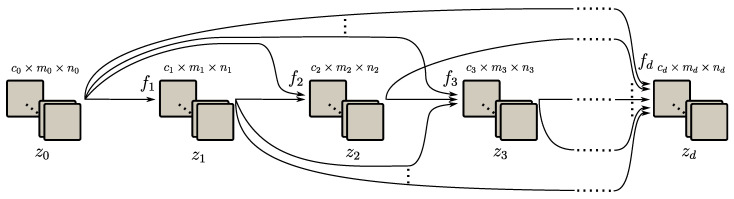
Schematic architecture of a neural network with dependencies between all layers. Squares denote feature map channels and arrows denote function inputs. The network depth is given by *d*.

There also exist more intricate neural network architectures (for instance in [18,19]) where the feature map $z_{i}$ in layer *i* can be written as function depending on the feature maps in all previous layers: $z_{i}=f_{i}\left(z_{0},z_{1},\ldots ,z_{i-1}\right)$, with $f_{i}:R^{c_{0}\times m_{0}\times n_{0}}\times \ldots \times R^{c_{i-1}\times m_{i-1}\times n_{i-1}}\to R^{c_{i}\times m_{i}\times n_{i}}$. This is schematically shown in Figure 2.

The network is parameterized with weights and biases which are typically involved in the functions $f_{i}$. The entire network can the be written as a function $F_{\theta }:R^{c_{0}\times m_{0}\times n_{0}}\to R^{c_{d}\times m_{d}\times n_{d}}$, where θ∈Θ contains given values for all weight and bias parameters. Since the final feature map $z_{d}$ produced by the network contains probability maps, it is usually compared with the one-hot encoding of the target image, which marks a pixel in channel *i* as probability one if the target class label of that pixel is *i*, and zero otherwise. Denote the one-hot encoding function by *P*. The aim is now to find a set of parameter values θ such that $P\circ F_{s}$ is approximated by $F_{\theta }$.

For imaging, convolutional neural networks (CNNs) have proven successful. In these networks the functions $f_{i}$ are typically operations involving activation functions, bias functions, weighting functions and, by definition, convolutions. These functions depend on the previous feature maps $z_{i-1}$ only or on feature maps of all previous layers. The latter case is more general, in which we have the following:

(3) $$\begin{array}{c} f_{i}{\left(z_{0},\ldots ,z_{i-1}\right)}^{j}=\sigma \left(\sum_{l=0}^{i-1}\sum_{k=0}^{c_{l-1}}C_{ijkl}\left(z_{l}^{k}\right)+b_{ij}\right). \end{array}$$

Here σ:R→R is the activation function, $b_{ij}\in R$ are the bias parameters and $C_{ijkl}:R^{m_{l}\times n_{l}}\to R^{m_{i}\times n_{i}}$ is the convolution function (including convolution filters) from feature map channel *k* in layer *l* to feature map channel *j* in layer *i*. During training, the parameters that are being optimized are the biases $b_{ij}$ and the convolutional filters in the convolutions $C_{ijkl}$. For a CNN $F_{\mathrm{Net}}:R^{c_{0}\times m_{0}\times n_{0}}\to R^{c_{d}\times m_{d}\times n_{d}}$ with depth *d* and $c_{0}=N_{b}$ spectral inputs, similar to Equation (2), the loss function is minimized over the network parameters $\theta \in \Theta_{\mathrm{Net}}$, to obtain the network $F_{{\mathrm{Net}}_{\theta }}$:

(4) $$\begin{array}{c} \min_{\theta \in \Theta_{\mathrm{Net}}}\sum_{i=1}^{N^{\mathrm{train}}}L\left(F_{{\mathrm{Net}}_{\theta }}\left(x_{i}^{\mathrm{train}}\right),P\left(y_{i}^{\mathrm{train}}\right)\right). \end{array}$$

#### 3.1.3. Spectral Data Reduction

If the uncompressed hyperspectral image data are used directly as input for the CNN, the large data size results in prohibitively long training times [51] and memory requirements [42]. A potential solution to these possible issues can be found by employing a spectral data reduction method. This method can be viewed as a function $G:R^{N_{b}\times m\times n}\to R^{N_{r}\times m\times n}$ that acts on the data *x* and transforms it to G(x) in a lower-dimensional space with $N_{r}\ll N_{b}$. Given a chosen reduction function *G*, the aim is now to find a CNN $F^{\prime }:R^{N_{r}\times m\times n}\to R^{N_{c}\times m\times n}$ that segments the reduced data, such that $F^{\prime }\circ G$ segments the original data, minimizes (4), and therefore approximates $P\circ F_{s}$:

(5) $$\begin{array}{c} \mathrm{Find}\;a\;\mathrm{function}\;F^{\prime }:R^{N_{r}\times m\times n}\to R^{N_{c}\times m\times n}\;\mathrm{such}\;\mathrm{that}\;\left(F^{\prime }\circ G\right)\left(x_{i}\right)\approx P\left(y_{i}\right)\;\mathrm{for}\;\mathrm{every}\;i. \end{array}$$

Since the function $F^{\prime }$ has only $N_{r}$ inputs, the input data size is strongly reduced, reducing the size of the minimization problem (4) and decreasing memory requirements. If G(x) preserves the relevant features in the image *x* that are required for the segmentation task, the function $F^{\prime }$ that minimizes (4) could, in principle, be more easily found.

In this paper, a new approach is proposed to reduce the data to a very limited number of input feature map channels, without the need for parameter tuning or prior information about the problem and providing possible advantages such as higher processing speeds.

### 3.2. Learned Data Reduction Method

We will now introduce our proposed task-driven end-to-end Data Reduction CNN (DRCNN) approach. The key idea of the method is to include the data reduction in the problem as a neural network to approximate the function $P\circ F_{s}$:

(6) $$\begin{array}{c} \mathrm{Find}\;\mathrm{functions}\;\;\;\begin{array}{c} G:R^{N_{b}\times m\times n}\to R^{N_{r}\times m\times n}\\ F^{\prime }:R^{N_{r}\times m\times n}\to R^{N_{c}\times m\times n} \end{array}\;\;\;\mathrm{such}\;\mathrm{that}\;\left(F^{\prime }\circ G\right)\left(x_{i}\right)\approx P\left(y_{i}\right)\;\mathrm{for}\;\mathrm{every}\;i. \end{array}$$

**Figure 3:**
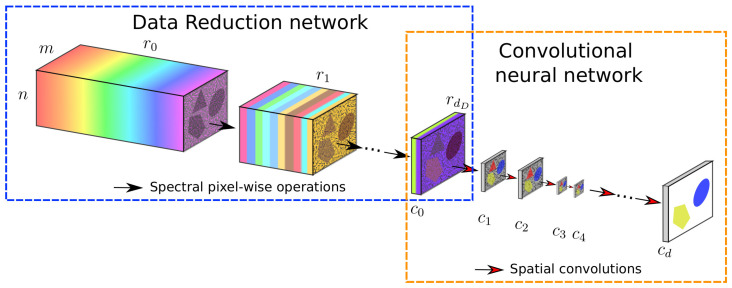
Schematic structure of a Data Reduction CNN example. The entire setup consists of a CNN of choice, for example with down- and upscaling layers as shown here, and a data reduction subnetwork in front. This subnetwork repeatedly decreases the number of images from $r_{0}$ down to $r_{d_{D}}$ by taking linear combinations of the input images. After that, the CNN carries out the segmentation task.

Therefore, the method includes a supervised data reduction tailored to the segmentation task, which can be separated from the CNN after being trained together with the CNN. The resulting new network is a combination of a subnetwork that spectrally reduces data to a given number $N_{r}$ of feature map channels and a CNN that segments the image from $c_{0}=N_{r}$ input feature map channels. A high-level overview of this approach is given in Figure 3. Given a CNN $F_{\mathrm{Net}}$, a compatible data reduction network is given by $G_{D}:R^{N_{b}\times m_{0}\times n_{0}}\to R^{N_{r}\times m_{0}\times n_{0}}$ with functions that consist of linear combinations of spectral feature map channels, as opposed to containing spatial convolutional operators as in the CNN. The data reduction layer is characterized by the data reduction list $D=[r_{0},r_{1},\ldots ,r_{d_{D}}]$, where $d_{D}$ is the depth of the subnetwork. The number of feature map channels $r_{i}$ in each layer dictates the data reduction, with $r_{0}=N_{b}$ and $r_{d_{D}}=N_{r}=c_{0}$. The feature maps ${\bar{z}}_{i}$ in each layer with i>0 are only dependent on those in the previous layer ${\bar{z}}_{i-1}$. Therefore, the architecture is of the form shown in Figure 1 (where the number of channels $c_{i}$ in layer *i* equals $r_{i}$ and the other spatial dimensions remain unchanged). As a result that the functions in this network are linear combinations (or equivalently, spectral pixel-wise 1×1 convolutions), similar to Equation (3), the functions $g_{i}:R^{r_{i-1}\times m\times n}\to R^{r_{i}\times m\times n}$ mapping the images from layer i−1 to *i* in the data reduction network have the form

(7) $$\begin{array}{c} g_{i}{\left({\bar{z}}_{i-1}\right)}^{j}=\bar{\sigma }\left(\sum_{k=1}^{r_{k-1}}{\bar{w}}_{ijk}\cdot {\bar{z}}_{i-1}^{k}+{\bar{b}}_{ij}\right). \end{array}$$

Here, $\bar{\sigma }:R\to R$ is the activation function in this subnetwork, ${\bar{w}}_{ijk}\in R$ are the linear weights between the *k*-th image in layer i−1 and the *j*-th image in layer *i*, ${\bar{b}}_{ij}\in R$ is the bias of image *j*-th image in layer *i*. The weights ${\bar{w}}_{ijk}$ and biases ${\bar{b}}_{ij}$ in these functions determine the set of parameters ϕ that has to be optimized in this data reduction subnetwork. For neural networks, the Rectified Linear Unit (ReLU) function is a commonly used activation function, but it can lead to dying nodes that become inactive and whose activation functions only output zeros once the nodes produce negative output values [52]. Since the final layers in the data reduction subnetwork contain a low number of feature map channels (at times only one), it is more likely that dying nodes will affect the performance of the network more negatively than in other network architectures. Therefore, for activation function $\bar{\sigma }$, we propose to use Leaky ReLU functions in the data reduction subnetwork with leakage parameter a=0.01 in order to avoid the dying ReLU output problem [52].

For this subnetwork, linear layers are used instead of convolutional layers, because we want to compress exclusively in the spectral direction and convolve exclusively in the spatial directions. Since adjacent spectral bins are highly correlated, applying local spectral convolutional operations is not expected to result in informative feature maps. Instead, bins that are more distant from each other should be combined to achieve this. A network with linear combinations is therefore more suitable than a convolutional neural network for learning this transformation as it can learn complex non-linear functions to combine the information from all spectral bins.

Let $\Theta_{G_{D}}$ denote the parameter space in this data reduction subnetwork $G_{D}$, compatible with the given CNN $F_{\mathrm{Net}}$. The optimization problem for the joint data reduction and image processing becomes

(8) $$\begin{array}{c} \min_{\begin{array}{c} \theta \in \Theta_{F_{\mathrm{Net}}}\\ \phi \in \Theta_{g_{D}} \end{array}}\sum_{i=1}^{N^{\mathrm{train}}}L\left(F_{\mathrm{Net},\theta }\left(G_{D,\phi }\left(x_{i}^{\mathrm{train}}\right)\right),P\left(y_{i}^{\mathrm{train}}\right)\right). \end{array}$$

The number of parameters to be trained in the data reduction layer is equal to $\left|\phi \right|=\sum_{i=0}^{d_{D}}r_{i}\cdot r_{i+1}+r_{i}$. The number of weights in the first layer of the data reduction network provides the leading order of the number of trainable parameter in this subnetwork. As the number of parameters in a CNN can be in the order of millions, the number of parameters in the data reduction layers is relatively small. Moreover, since the CNN has fewer input images, depending on the architecture, the data reduction network may also reduce the number of parameters in the CNN, possibly making a pass through the network faster.

## 4. Experiments and Results

### 4.1. Data Reduction Network Architectures

The proposed data reduction approach is designed to be compatible with any existing CNN. In this paper, we present results for both the popular U-Net CNN architecture [19] and the recent Mixed-Scale Dense (MSD) architecture [18]. Their architectures and the data reduction integration are explained first, after which the datasets, experiments and results on the datasets are outlined.

#### 4.1.1. Data Reduction Multi-Scale Dense Net

**Figure 4:**
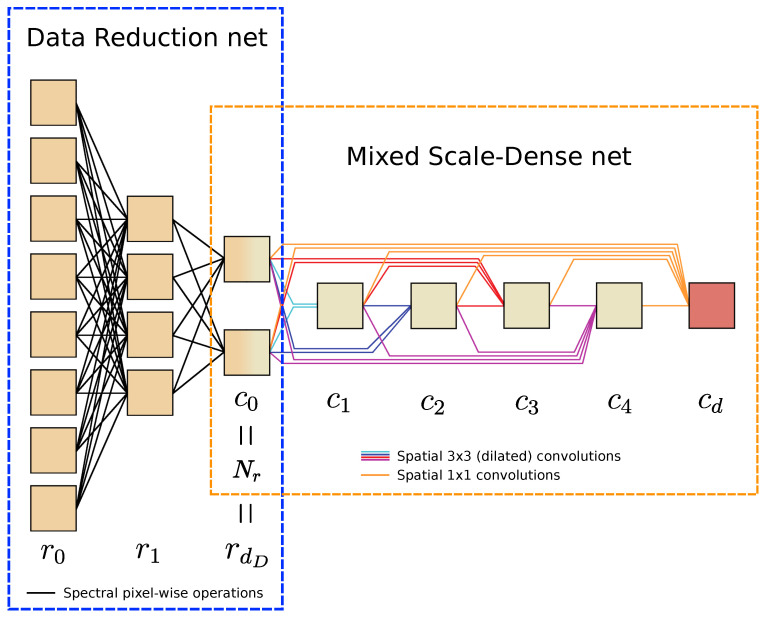
Example of a Data Reduction Mixed-Scale Dense (MSD) network structure. The number of channels are indicated with the feature maps. Since w=1 is chosen, $c_{i}=1$ for i>0. The data are reduced from $r_{0}=8$ input images to $r_{d_{D}}=N_{r}=c_{0}=2$ feature map channels in the data reduction net, while the segmentation task is performed by an MSD net of depth d=5. Each 3×3 convolution is followed by a Rectified Linear Unit (ReLU) operation.

In the MSD network, all features maps are fully-connected and the operations are dilated convolutions (also called atrous convolutions) to capture image features at different scales. As in the paper that introduced the MSD network structure [18], a network width of w=1 works well in our experiments (or equivalently, setting all values $c_{i}$ to 1 for i>0). Figure 4 gives an example of a Data Reduction MSD (DRMSD) net layout with a reduction to $N_{r}=2$ feature map channels, where the depth of the data reduction net is equal to $d_{D}=2$, the reduction scheme is D=[8,4,2] and the depth of the MSD net is d=5. In this paper, we use a common depth of d=100 for this MSD network.

The dilations in each convolutional layer range from 1 to 10, repeatedly increasing from 1 to 10 over the depth of d=100. All dilated convolutions are followed by a ReLU operation. All bias and weight parameters are initialized to zero. For the convolution weights, Xavier initialization is used. During training, ADAM optimization [53] is used on the cross-entropy loss between the data and the predictions. We use the CPU and GPU implementations in Python of [18,54], with additional CPU and GPU implementation for the data reduction component. Each network is trained on one GPU core of a GeForce GTX TITAN X with CUDA version 10.1.243.

#### 4.1.2. Data Reduction U-Net

**Figure 5:**
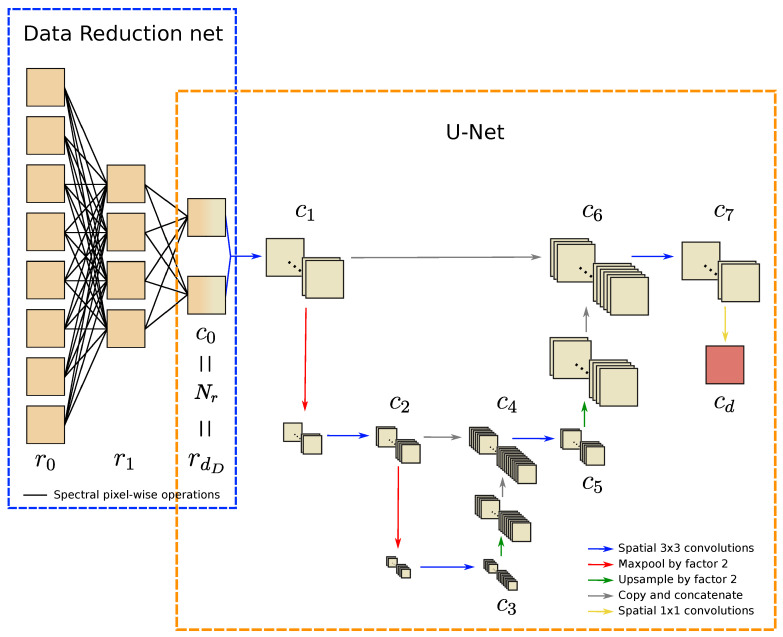
Example of a Data Reduction U-Net structure. The number of channels are indicated with the feature maps. The data are reduced from $r_{0}=8$ input images to $r_{d_{D}}=N_{r}=c_{0}=2$ feature map channels in the data reduction net. Each 3×3 convolution is followed by a ReLU operation. The number of channels is shown after each convolution and concatenation operations. The network is designed to have $c_{1}=c_{7}$, $c_{2}=c_{5}=2c_{1}$, $c_{3}=4c_{1}$, $c_{4}=6c_{1}$ and $c_{6}=3c_{1}$. The value of $c_{d}$ is equal to the number of segmentation classes |C|.

A second CNN architecture that is used for the experiments is the commonly used U-Net. An example of a Data Reduction U-Net (DRUNet) with reduction scheme D=[8,4,2] is given in Figure 5. In this example, the data are reduced to $N_{r}=2$ feature map channels, which in turn is the input for the U-Net subnetwork. In the U-Net architecture used in our experiments, the feature maps are downsampled twice, with a stride of 2, and the initial number of feature map channels is $c_{1}=128$. Bilinear interpolation is used for upsampling. The number of feature map channels doubles in each downsampling layer, which gives $c_{2}=c_{5}=256$, $c_{3}=512$, $c_{4}=768$, $c_{6}=384$ and $c_{7}=c_{1}=128$. All downsampling and upsampling operations are preceded and followed by a spatial 3×3 convolution operation with zero padding, each of which is followed by a ReLU activation function. All biases and weights in the data reduction layers are initialized to zero, whereas the biases and convolution weights are initialized by sampling from $U(-\sqrt{k},\sqrt{k})$, where $k=\frac{1}{c_{\mathrm{in}}\cdot a^{2}}$ is the range, $c_{\mathrm{in}}$ is the number of input channels and *a* is the kernel size. During training, ADAM optimization is used on the average of the binary cross entropy loss and the dice loss [55,56] between the data and the predictions. The network is implemented using PyTorch [57,58] and is trained on one GeForce GTX TITAN X GPU core with CUDA version 10.1.243.

### 4.2. Datasets

In this section, we will introduce the datasets that are used in the experiments. Hyperspectral data reduction methods are commonly compared using satellite datasets which consist of one hyperspectral image of a certain location (Pavia University, Indian Pines, and Salinas for example [59]), with annotated ground truth segmentation values, some of which are very rare in the image. The use of only one image may cause information leaks between training and test sets when evaluating convolutional neural networks on the same image [17], since overlapping spatial information is likely to be used for classifying pixels in both sets. The availability of other hyperspectral datasets with multiple labeled training samples is limited [11,60]. We opt to use generated artificial hyperspectral X-ray and remote sensing datasets to resolve this problem, since they consist of multiple images that can be divided into different independent sets.

#### 4.2.1. Simulated Attenuation-Based Hyperspectral X-ray Dataset

**Figure 6:**
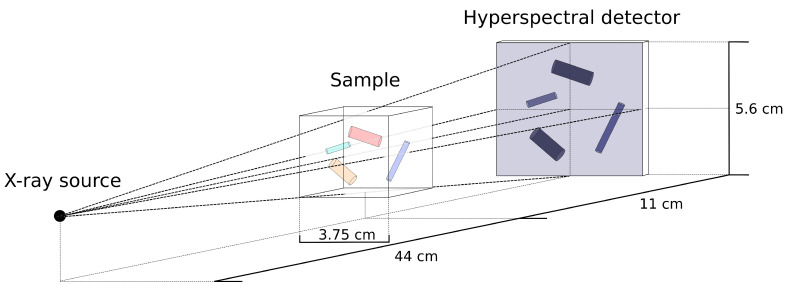
Schematic overview of the hyperspectral X-ray projection setup with a cone beam geometry.

**Figure 7:**
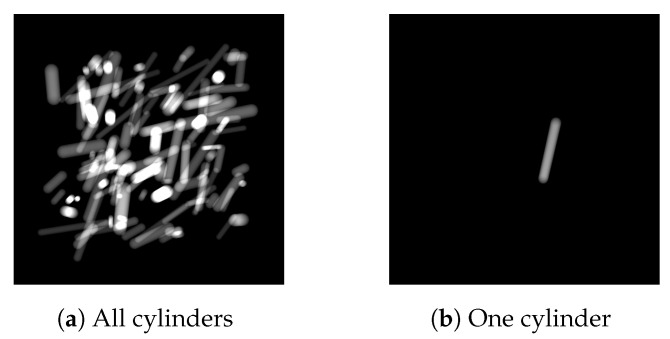
Example of the simulated material projections before material designation. The cylinders are shown to be all combined in one image (**a**), and separately (**b**).

The first simulated dataset on which we test the method is based on the physical properties in hyperspectral X-ray imaging, including the geometric setup, source spectrum and attenuation properties of various materials. We leave out other effects such as scattering and detector responses as they do not substantially contribute to the understanding of the data reduction network properties. The dataset contains 100 2D images of size 512×512 consisting of $N_{b}=300$ spectral bins each. These are simulated X-ray projections of 3D volumes of 1024×1024×1024 voxels containing 120 cylinders with randomized lengths, thicknesses, angles and positions. A schematic overview of the simulated X-ray setup is given in Figure 6. A virtual source and a virtual detector of size 1536×1536 are placed in front and behind the object, respectively, and we use the ASTRA toolbox [61,62] to compute the projections of size 512×512 from this geometric setup. An example of a projection of 120 cylinders and one cylinder is given in Figure 7.

**Figure 8:**
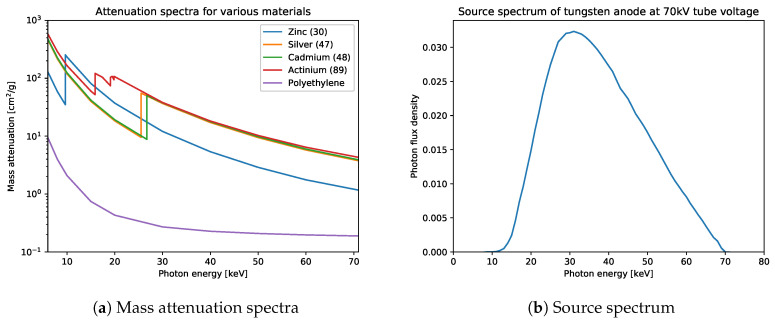
Mass attenuation spectra for zinc, silver, cadmium, actinium and polyethylene from 6 keV to 71 keV (**a**). In this spectral region, zinc, cadmium and actinium have one K-edge, polyethylene has none, while actinium has multiple edges. Note that the K-edges of silver and cadmium are relatively close to each other. This holds for all adjacent atomic numbers (not shown in this figure). (**b**) The normalized plot of the source spectrum ${\bar{I}}_{0}$ used for generating the hyperspectral X-ray projections.

For the experiments, we assign materials to these 120 cylinders, by means of assigning atomic numbers in two different setups. In the first few-material setup, we assign atomic number 47 (silver) to two randomly chosen cylinders whereas the remainder is assigned 48 (cadmium). In the second many-material setup, each material from atomic numbers 30 (zinc) up to 89 (actinium) is uniquely assigned to two randomly chosen cylinders. To prevent the cylinders with high atomic numbers to be too highly attenuating, the cylinders consist of a mix of 99% polyethylene and 1% of the assigned material. An overview of a selection of the attenuation spectra is given in Figure 8a. The spectra are taken from the National Institute for Standards and Technology (NIST) [63]. Further details on the setup and on computing the projections are given in Appendix A.

**Figure 9:**
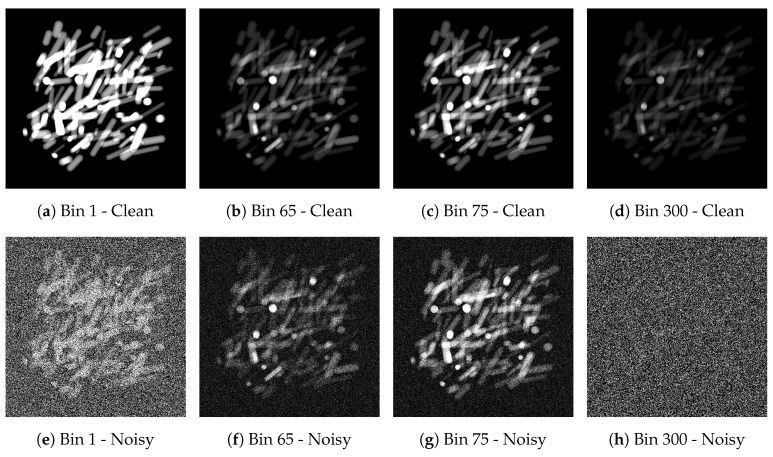
Visualization of the simulated X-ray data at different bins. The K-edge transition of cadmium is visible between bins 65 and 75 (among (**a**)–(**d**), compare (**b**,**c**) for example). The data in bins 1 and 300 (**e**,**h**) are much more noisy than in bins 65 and 75 (**f**,**g**), due to low source spectrum values at bin 1 and 300.

Poisson noise is applied to both the projection images and the flatfield images, i.e., projection images without objects. In this case, the flatfield images are averaged over 50 separate flatfield images. As a result of the shape of the source spectrum ${\bar{I}}_{0}$, shown in Figure 8b, the flux of photons is lowest at low and high energies. Therefore, the bins corresponding to energies close to 13 kV and 70 kV are more noisy than the others. Example images of noisy and clean data from the few-material datasets are given in Figure 9. We combine the clean and noisy setups with the few-material and the many-material settings, resulting in four combinations of datasets. The data are 31.5 GB in size for every combination.

#### 4.2.2. Simulated Reflectance-Based Hyperspectral Remote Sensing Dataset

**Figure 10:**
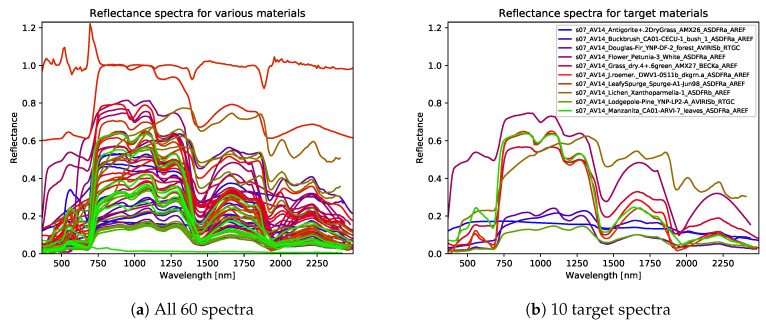
Reflectance spectra used for this dataset (**a**). The 10 target spectra on the right (**b**) are a subset of the 60 spectra. The filenames of these target spectra in the USGS Library are added.

In addition to the previous dataset, we created a simulated dataset where the spectral properties are taken from remote sensing settings. This dataset again contains 100 2D images of size 512×512, now consisting of $N_{b}=200$ spectral bins. We create 360 cylinders of different sizes and place these in the images such that none of these overlap. Each of these cylinders is assigned a material in such a way that there are 60 different materials with 6 cylinders each. The reflectance spectra are taken from the United States Geological Survey (USGS) High Resolution Spectral Library [64,65], which contains a wide variety of reflectance spectra for liquids, minerals, soils and vegetation, among other categories. The spectra used for this dataset (Figure 10a) are randomly drawn from the vegetation section. For the experiments, we consistently choose 10 labels out of the 60 which need to be detected. The spectra of these materials are given in Figure 10b. The spectral range we use is from 450 nm to 2400 nm, and we only use materials for which the full spectrum in this range is included in the library.

**Figure 11:**
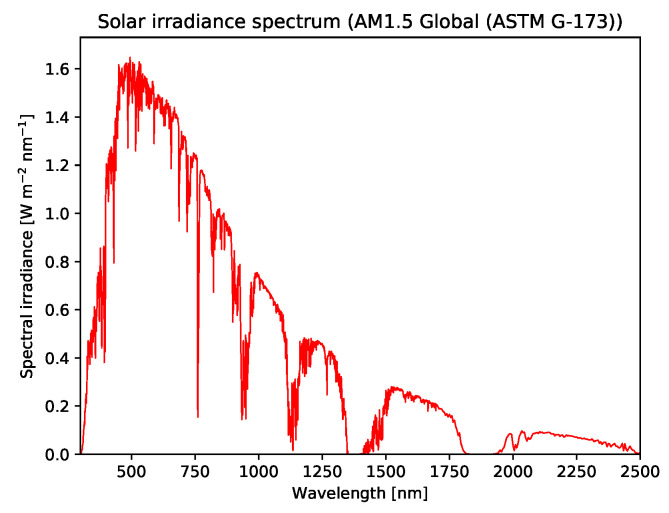
The solar irradiance spectrum used for the remote sensing experiments. Note that the drops to a value close to 0 in the graph, particularly at wavelengths 1350–1400 nm and 1800–1950 nm, are mostly due to absorption by water vapor.

The reflectance spectra are multiplied by the solar irradiance spectrum, which is the base intensity received from the Sun. We use the spectrum AM1.5 (G-173-03 International standard) global [66] from the American Society for Testing and Materials (ASTM) [67] that gives terrestrial solar spectral irradiance on a surface under certain conditions such as orientation towards the Sun, temperature, pressure and atmosphere composition, among other conditions [66]. The solar irradiance spectrum is given in Figure 11. At certain wavelengths there is low transmittance through the atmosphere of the Earth due to presence of certain substances, for instance carbon dioxide, oxygen and most importantly water vapor. On the resulting images, we apply Gaussian noise where the standard deviation is $\frac{1}{1000}$ of the maximum signal in the dataset. The resulting images are the measured signals. There are many ways to normalize the radiance data, and we apply a flatfield correction [68] using the solar irradiance spectrum given in Figure 11. The bins located in regions where solar irradiance is blocked have a very low signal-to-noise ratio.

**Figure 12:**
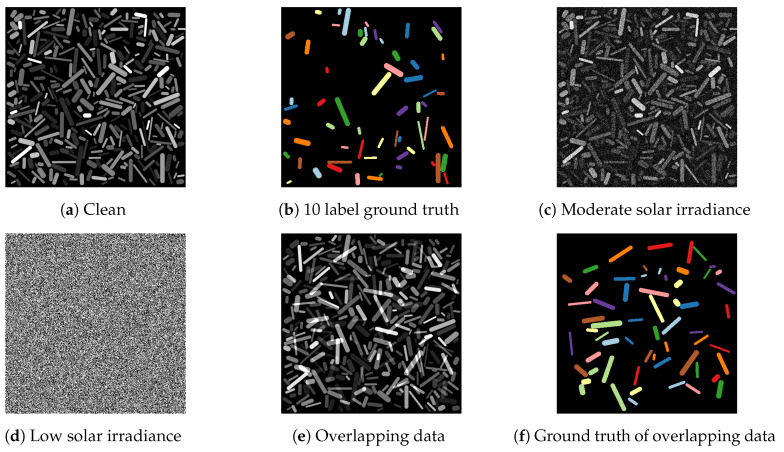
Visualization of the simulated remote sensing data. The clean data and the ground truth are shown in (**a**,**b**), respectively. When the described Gaussian noise is added to this data, many bins still resemble the clean data, but (**c**) shows a moderately noisy bin and (**d**) shows an extremely noisy bin, resulting from differences in solar irradiance. The data with overlapping cylinders and their representation as ground truths are given in (**e**,**f**).

In addition to the data described above, we created a dataset where the reflectance images of the 10 target materials are imposed on those of the 50 remaining materials. This creates overlap between these two material sets but keeps the materials within these sets non-overlapping. This simulates mixed material reflectance signals that are likely to occur in realistic remote sensing data. Figure 12 shows visualized examples of the data. Note that the difference with the hyperspectral X-ray dataset is that we now have 11 classes instead of 2 to classify pixels into.

### 4.3. Implementation of Standard Data Reduction Methods

In the experiments, we will compare the proposed data reduction method with standard data reduction methods (PCA, NMF and LDA). These methods are implemented using Scikit-learn (version 0.22.1) [69]. In all cases, the default settings have been used. For memory limitation reasons, a subset of the data points is used to compute each transformation, where every *k* data points in the images are included. For computing the standard data reduction transformation, we sample every k=2 data points when employing PCA, every k=5 for NMF and every k=6 for LDA. When reducing to 200 feature map channels, we use k=3 data points for PCA, k=6 for NMF and k=6 for LDA.

### 4.4. Results

In this section, we describe all experiments, and outline the results. For each experiment, we use $N^{\mathrm{train}}=70$ images for training, $N^{\mathrm{val}}=20$ for validation and $N^{\mathrm{test}}=10$ for testing from the relevant datasets. During training, data augmentation is applied by rotating and flipping the input images, resulting in a total of 70 × 8 = 560 training images. The chosen training time of MSD is 2 days on the hyperspectral X-ray data (ranging from ca. 300 to 6500 epochs), and 3 days on the generated remote sensing data (ranging from ca. 1500 to 9000 epochs), based on the results on the validation sets. After these time durations most networks did not show any more improvement on the validation sets. The training lengths for U-Net are fixed to 3000 epochs (roughly 1.5 days on average), as the U-Nets converged faster. Despite the possibility to employ multilayered data reduction networks, we experienced that a depth of $d_{D}=1$ yielded the best segmentation results. The additional advantage of this is that tuning the sizes of intermediate data reduction layers can be avoided. In addition, the number of trainable parameters and the processing and training times are also slightly lower as a consequence. For all experiments, we take the network with the best performance on the validation set and measure its average class accuracy over all 10 segmentation images in the test set. The average class accuracy is the average number of correctly classified pixels per class relative to the total number of pixels in that class, given by:

$$\begin{array}{c} \frac{1}{\left|C\right|}\sum_{c\in C}\frac{TP_{c}}{TP_{c}+FN_{c}} \end{array}$$

Here, C is the set of classes, $TP_{c}$ is the number of correctly classified (true positive) pixels with true class *c* and $FN_{c}$ is the number of incorrectly classified (false negative) pixels with true class *c*. To test the robustness of our method, we present averages and standard deviations over 8 runs for a few selected experiments in Appendix B.

#### 4.4.1. Noise and Multiple Materials

For the assessment of the segmentation accuracies of DRCNN compared to those using other data reduction methods, we apply reduction to $N_{r}=2$ feature map channels while varying the data reduction method on both the simulated hyperspectral X-ray dataset and the remote sensing datasets. To determine what properties of the data contribute to the performance of the methods, we vary the inclusion of noise and the number of materials (2 or 60) for the X-ray dataset (Section 4.2.1). For the remote sensing dataset, we vary the inclusion of noise and overlapping materials (Section 4.2.2). MSD and U-Net networks without data reduction are trained on the full hyperspectral data as well. Note that since there are only two target classes in the X-ray dataset, LDA will reduce the data to one channel.

The results of the experiments on the X-ray datasets and the remote sensing datasets are summarized in Table 1 for MSD and U-Net. All data reduction methods obtain high accuracy (>99%) in the case of clean data and two materials. However, PCA and NMF obtain a significantly lower accuracy when multiple materials are introduced (<83%), while LDA, DRCNN and CNN without reduction retain the high accuracy (>99%). For two-material data, when noise is introduced, the average class accuracies for NMF, PCA and to a lesser extent LDA decrease (<91%), while DRMSD, DRUNet, MSD and U-Net still maintain high accuracy (>98%). This difference for LDA is amplified with MSD when dealing with many materials in a noisy setting, with LDA having a notably lower accuracy than in the two-material setting (at ca. 76% accuracy), although this trend is not seen with U-Net (ca. 85%). The other two data reduction methods have a significantly reduced avaraged class accuracy (<58%). Both DRMSD and DRUNet have a high performance (>98.5%) in this case, showing that they are robust to both noise and the inclusion of multiple materials. The experiments on the remote sensing datasets show a similar trend. Table 1 shows that the robustness of DRMSD and DRUNet remains (ca. 98% or higher), but the differences with LDA when including noise are smaller (>90%). For PCA and NMF the accuracy decreases only slightly when overlap is included, but does decrease significantly when noise is introduced (down to ca. 9%). To summarize, DRMSD and DRUNet are shown to be able to remain mostly robust to noisy and multiple materials (ca. 98% or higher accuracies) in these simulated datasets.

#### 4.4.2. Number of Reduction Feature Map Channels

To assess the image quality of the DRCNN as a function of the number of spectrally reduced feature map channels, we carried out experiments for both datasets. For the X-ray dataset, we use 1, 2, 10, 60 and 200 feature map channels. For the remote sensing dataset we focus on small feature maps, using 1, 2 and 10 channels. For the latter dataset, we also vary the properties of the dataset (clean/noise and overlap) to assess the influence of those properties on the performance, and a layered reduction to 2 and subsequently 1 map is added. For comparison, the performance using the other standard data reduction methods is also assessed, and for reduction of LDA to more than one feature map channel with the X-ray data, we add prior knowledge about the presence of other materials in the ground truth. For reduction to 2 feature map channels, the 59 other materials are added as one additional class. For reduction to 10 channels, these other materials are added as 9 classes consisting of 6 to 7 materials each, grouped by their atomic numbers. For reduction to 60 channels, each remaining material is added as a separate class. Note that this prior knowledge is in many practical cases not available, so this constitutes an artificial comparison.

**Figure 13:**
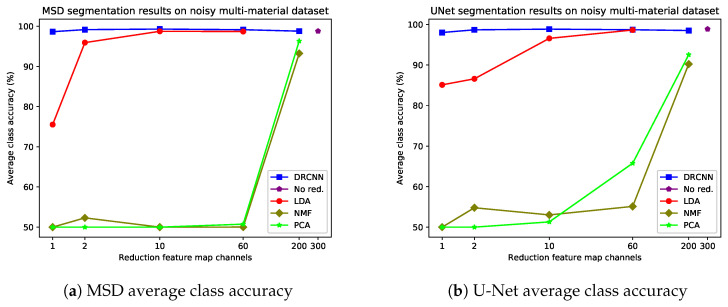
Average class accuracies for different data reduction methods using MSD (**a**) and U-Net (**b**) on the noisy multi-material X-ray dataset. As a reference, the results for standard MSD and U-Net net are included, which act directly on all 300 spectral bins.

Figure 13 outlines the results of the experiment for the noisy multi-material X-ray dataset. First of all, DRMSD and DRUNet have high average class accuracies for all number of reduction feature map channels. The accuracy is highest when reducing to 2 or 10 feature map channels, and the reduction to only 1 feature map channel is only slightly lower in comparison. In any case, all accuracies for DRMSD and DRUNet are higher than 98%. By contrast, PCA and NMF reach an accuracy of more than 90% when 200 or more reduction map channels are used, but for 60 or fewer reduction channels the accuracies remain below 70%, showing that both data reduction methods are not suitable when 60 or fewer channels are required to reduce the data to. For LDA, the data are reduced to 1 feature map channel and the accuracy is lower than both DRMSD and DRUNet (<85%). It is only when prior knowledge about the 59 other materials is included that the accuracy approaches that of the DRCNN methods, and even then it may only attain a similar accuracy when the data are reduced to more than 2 feature map channels.

**Figure 14:**
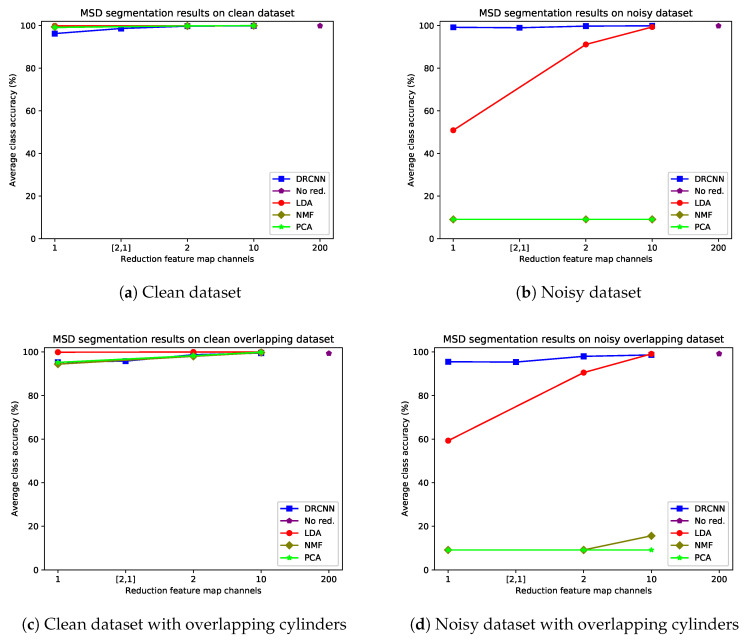
Average class accuracies for different reduction schemes with MSD as CNN for different simulated remote sensing datasets: clean dataset (**a**), noisy dataset (**b**), clean overlapping dataset (**c**) and noisy overlapping dataset (**d**). The layered reductions to 2 and then 1 feature map channel(s) are indicated by [2,1].

**Figure 15:**
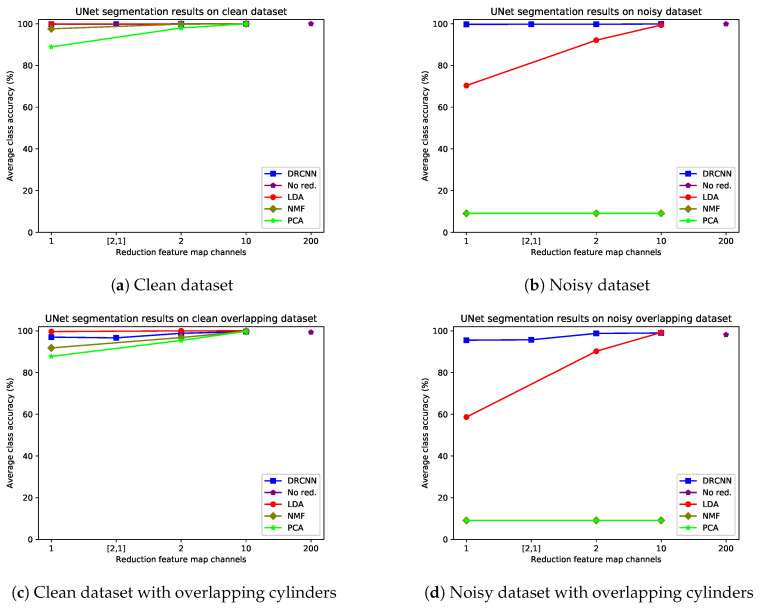
Average class accuracies for different reduction schemes with U-Net as CNN for different simulated remote sensing datasets: clean dataset (**a**), noisy dataset (**b**), clean overlapping dataset (**c**) and noisy overlapping dataset (**d**). The layered reduction to 2 and then 1 feature map channel(s) is indicated by [2,1].

**Figure 16:**
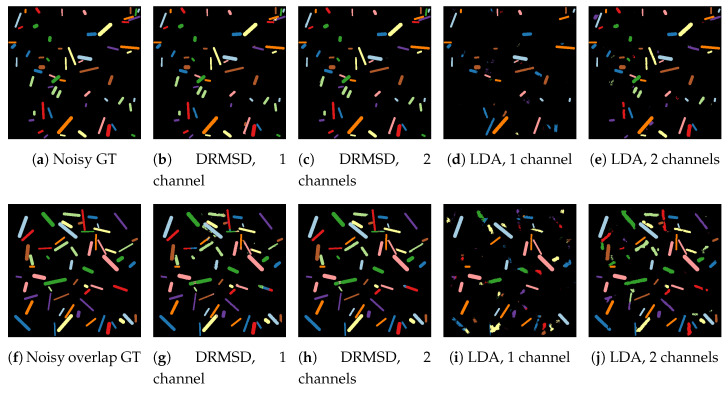
Visual results for LDA and DRMSD reduction schemes for reductions to 1 and 2 feature map channels on the noisy dataset (**a**–**e**) and the noisy overlapping dataset (**f**–**j**).

For the remote sensing datasets, the quantitative segmentation results of applying the trained networks to the test sets for DRMSD and DRUNet are given in Figure 14 and Figure 15 respectively, broken down into the four combinations of noise and overlap properties. The visual results for MSD are given in Figure 16. DRMSD and DRUNet show high accuracies (>99%) on the standard noisy dataset for all number of reduction map channels, but when the target cylinders are overlapping other cylinders, the accuracies for DRMSD and DRUNet become slightly lower (about 95.5%) when compressing to one feature map channel. In this case, both DRCNNs do not miss any cylinders but there are artifacts on some detected cylinders. However, when the number of reduction feature map channels increases, the accuracy increases rapidly (to ca. 98% and higher). The layered reduction D=[2,1] images show no strong additional value over direct reductions to 1 image in any experiment. On the other hand, the LDA accuracy is considerably lower for reductions to 1 or 2 feature map channels for all noisy datasets (<70% and <93%, respectively) and reaches a comparable accuracy with reductions to between 3 and 10 feature map channels. From the visual results it can be observed that segmentation from LDA reduced data causes the network to completely miss certain cylinders. In all experiments with noisy data PCA and NMF, the accuracy is ca. 9%.

We conclude that for both datasets and most of the properties we investigated, the image quality of the DRCNNs is acceptable for reductions to one feature map channel and higher accuracies are attained when the number of reduction feature map channels is slightly increased. The methods perform favorably compared to the common data reduction methods.

#### 4.4.3. Dependence of Feature Map Properties on Data Reduction Method

In the final experiment, we look at the properties of trained networks on the datasets when the number of reduced feature map channels is set to $N_{r}=1$. For the X-ray dataset, we compare the feature maps with standard reduction images and assess whether the network makes use of the distinguishing attenuation properties (Figure 8a) of the materials to identify the target cylinders. Along with the X-ray dataset, we look at the weights as a function of spectral bins, to assess the differences in learned weights by MSD and U-Net.

**Figure 17:**
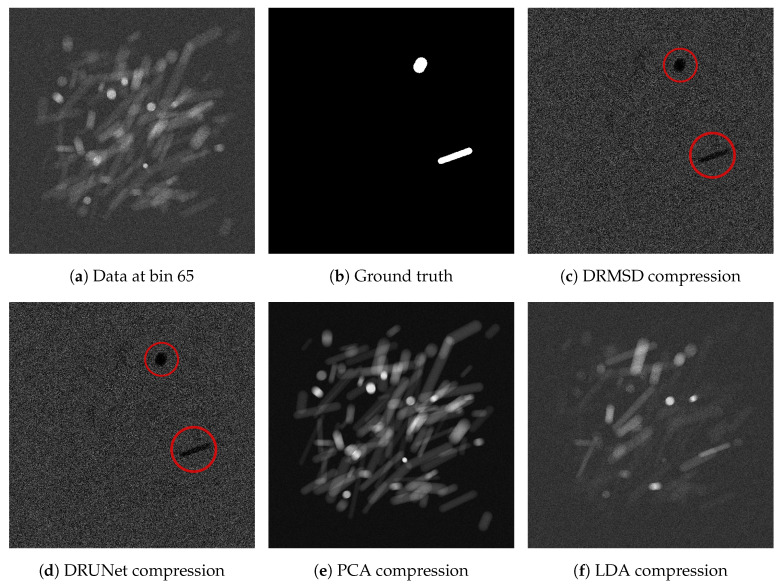
Visual comparison of the data reduction methods for reduction of the noisy many material dataset (**a**,**b**) to 1 image. Despite the high noise, DRMSD (**c**) and DRUNet (**d**) create the most distinctive images with respect to the ground truth (note the dark shapes at the target cylinder locations, indicated by red circles). The PCA (**e**) and LDA (**f**) compressions are included, but the Nonnegative Matrix Factorization (NMF) compression is omitted, as it is highly similar to the PCA compression.

**Figure 18:**
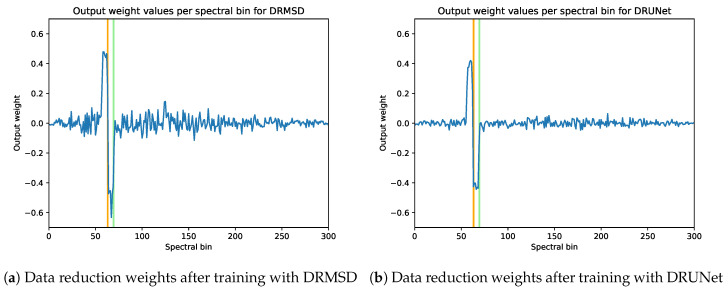
Data reduction weights per bin after training with DRMSD (**a**) and DRUNet (**b**) on the noisy few-material X-ray dataset. The K-edge of the material of the objects to be detected (silver) is located between bins 63 and 64 (indicated in orange), which is the location of the drop. The K-edge of cadmium is indicated in green. Additionally, note that the absolute value of the weights decreases when approaching bin 1 or 300.

Figure 17 shows the reduction images relative to the ground truth, for all different reduction methods (including DRMSD and DRUNet) to one feature map channel. While the standard reduction methods do not yield very distinctive reductions, the DRMSD and DRUNet methods give a reduction that, although noisy, already gives a clear indication in black what the locations of the ground truth objects are. In Figure 18a, the output weights for the first layer in the DRMSD network are plotted as a function of the energy bins, which gives an indication of what the network learns during the training process. First of all, there is a clear peak at bins 60–63 and a valley at bins 64–67. The K-edge transition of the ground truth material silver is located between bins 63 and 64. Since the attenuation of silver changes between these bins, the network learns to take combinations of bins with energy slightly lower than that of the edge and bins with energy slightly higher than that of the edge, with positive and negative weights, respectively, to make the silver objects stand out. The other bins have decidedly lower weight magnitudes that revolve around zero, roughly canceling out their contribution to the compressed image. It shows that the network can learn that only the aforementioned bins are critical to performing this segmentation task. Additionally, the weights become even smaller when bin 1 or 300 is approached, showing that the network learns to disregard very noisy bins. In Figure 18b the same quantities are plotted for a DRUNet trained network. The peaks and sharp transitions are visible in this case as well, but the absolute values of the non-peak weight values are generally lower than those of DRMSD.

**Figure 19:**
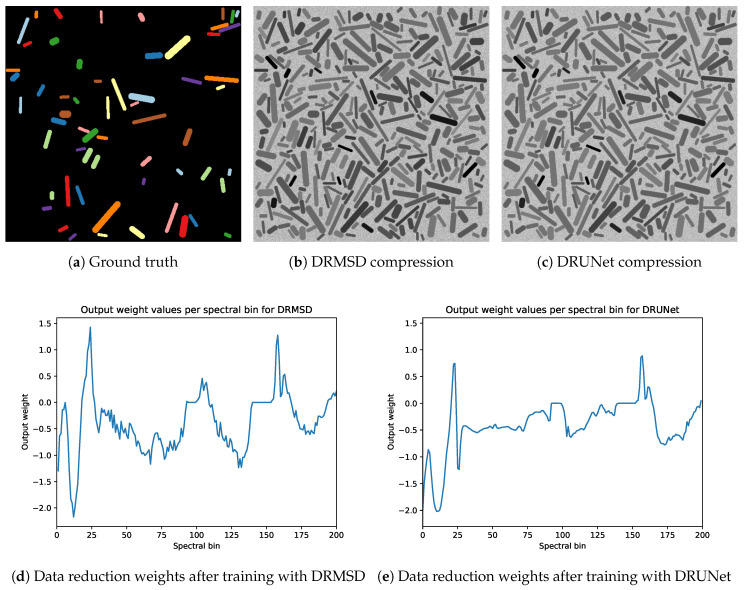
Example of data reduction weights (**d**,**e**) and resulting compressions (compare with ground truth (**a**)) for DRMSD (**b**) and DRMSD (**c**) with reduction to one feature map channel for the noisy remote dataset without overlap.

For the noisy remote sensing dataset without overlap, Figure 19 shows an example image with their corresponding DRMSD and DRUNet compression and weight values of in the data reduction layer when reducing to one feature map channel. While the shape of the graphs are different, there are some similarities. First of all, the weights of the bins 94–98 and 141–153, corresponding to the wavelengths from which noisy data arises, are zero. Thus, both networks learn to leave out noisy bins. Apart from this, despite the differences in network architecture, there are some similarities in the shapes of the graphs of the weights, mostly in the bin ranges 0–30 and 141–200.

## 5. Discussion

We have applied the proposed Data Reduction CNN approach to two simulated datasets. We expected these datasets to be challenging since they contain noisy bins that have to be identified and left out. Furthermore, for the X-ray dataset one material has to be identified among 60 others, all of which may be superimposed on the same location in the projection images. For the remote sensing dataset, we expected that high spectral similarities between materials make identifying 10 different classes simultaneously a challenging task, especially when reducing to only one or two feature map channels. The Data Reduction CNN is able to obtain high accuracy with the proposed approach of simultaneous data-driven compression and training (Section 4.4.1). Note that we observe some slightly higher accuracies for DRCNN compared to the standard CNN approach in some cases and some slightly lower accuracies in other cases. We expect that this is because of statistical deviations due to the random nature of training CNNs. As can be seen in Section 4.4.3, the data reduction subnetwork learns to map spectral properties of the relevant materials to reduction feature maps, while the CNN simultaneously extracts the spatial properties from these feature maps for accurate segmentation. Whereas other common data reduction methods are expected to need considerably more feature map channels for a high accuracy, the DRCNN method is able to compress the data without any (hyper)parameter tuning to a very limited number of feature map channels (Section 4.4.2). The data reduction layer can, in principle, be successfully combined with any CNN, provided that this CNN without data reduction can also solve the imaging task.

Eventually, the compression method can be easily extracted from a trained network, such that the compression procedure and the classification task can be carried out at separate locations. In addition, the training procedure has to be carried out only once after which the task it has been trained for can be performed at high-speed throughput. Depending on the CNN architecture, the data reduction approach may speed up the training and application process as the reduction to few feature map channels may significantly decrease the number of trainable parameters in the network.

In future work, we plan to apply this approach to real datasets and in practical problem settings and assess whether the satisfactory accuracies and robustness results (Appendix B) carry over. The data reduction method shows accurate preliminary results on common benchmark datasets such as Pavia University, Indian Pines and Salinas. Therefore, it will be interesting what DRCNN can achieve on large and challenging experimental hyperspectral datasets.

## 6. Conclusions

In this work, we have proposed a task-driven end-to-end approach for supervised deep learning in hyperspectral imaging problems by adding a data reduction component to a convolutional neural network. The method is designed to work with any CNN and the combined Data Reduction CNN (DRCNN) network learns to effectively spectrally reduce the data for a given task, without the need for prior knowledge or network and parameter tuning. The data reduction subnetwork is directly connected with the chosen CNN and learns to combine data from hyperspectral bins, which is done simultaneously with training of the CNN for the imaging task. Using a simulated hyperspectral X-ray dataset and a simulated hyperspectral remote sensing dataset, we have demonstrated with a Multi-Scale Dense (MSD) and a U-Net network that a DRCNN can learn complex reductions from a typically large spectral dimension to a very limited number of feature map channels. As opposed to standard reduction methods such as PCA, NMF or LDA, this learned data reduction method finds essential distinctive task-specific features in the hyperspectral data while retaining high imaging task accuracies when compressing these features into a very limited number of feature map channels. We have shown that, despite noise and the presence of multiple overlapping material properties, high compression can be achieved with Data Reduction CNNs, resulting in significant advantages for high-compression and high-throughput applications.

## Figures and Tables

**Table 1:** Average class accuracies for various datasets and data reduction methods for reductions to two feature map channels (except for Linear Discriminant Analysis (LDA) on the X-ray dataset, which is reduced to one feature map channel, since there are only two target labels). Accuracies below 97.5% are indicated in italic red, and the highest value(s) per row for each dataset and CNN are indicated in bold.

			MSD					U-Net				
			**PCA**	**NMF**	**LDA**	**DRCNN**	**No Red.**	**PCA**	**NMF**	**LDA**	**DRCNN**	**No Red.**
**X-ray**	Clean	2 mat.	99.70	**99.73**	**99.73**	**99.73**	99.37	99.66	99.67	99.67	99.69	**99.72**
		60 mat.	*60.36*	*82.69*	**99.69**	99.68	99.42	*79.64*	*82.92*	**99.72**	99.69	99.69
	Noisy	2 mat.	*50.00*	*57.31*	*90.39*	**99.11**	98.60	*50.00*	*50.00*	*79.49*	**98.92**	98.40
		60 mat.	*50.00*	*52.31*	*75.53*	**99.16**	98.77	*50.00*	*54.79*	*85.11*	98.69	**98.86**
**Remote sensing**	Clean	No overlap	99.87	99.85	**99.94**	99.75	99.90	97.97	99.81	99.87	99.95	**99.97**
		Overlap	98.29	97.99	**99.97**	98.74	99.33	*95.39*	*96.78*	**99.98**	98.82	99.28
	Noisy	No overlap	*9.09*	*9.09*	*91.15*	99.76	**99.86**	*9.09*	*9.09*	*92.10*	99.76	**99.87**
		Overlap	*9.09*	*9.09*	*90.53*	97.98	**99.17**	*9.09*	*9.09*	*90.20*	**98.76**	98.13

## Abbreviations

The following abbreviations are used in this manuscript:

CNN	Convolutional neural network
DRCNN	Data Reduction CNN
PCA	Principal Component Analysis
NMF	Nonnegative Matrix Factorization
LDA	Linear Discriminant Analysis
MSD	Mixed-Scale Dense (network)
DRMSD	Data Reduction MSD
DRUNet	Data Reduction U-Net

## Appendix A. X-ray Projection Data Computation

In this appendix, we provide further details on the computation of the simulated X-ray projections. The dataset consists of 100 2D images of size 512×512 with $N_{b}=300$ spectral bins. These are simulated X-ray projections of 3D volumes of 1024×1024×1024 voxels containing 120 cylinders with randomized lengths (uniformly between 0.143 and 1.43 cm), thicknesses (uniformly between 0.044 and 0.11 cm), angles and positions. For a schematic overview of the simulated X-ray setup, we refer to Figure 6. A virtual source and a virtual detector of size 1536×1536 are placed in front and behind the object respectively, and we use the ASTRA toolbox to compute the projections from this geometric setup. After this, we downscale the projections to 512×512 for computational efficiency, effectively rescaling the volume size as well. The detector pixel size is chosen to be $s_{\mathrm{pixel}}=0.11$ mm, making the detector about 5.6 cm, while the voxel size is chosen to be $s_{\mathrm{voxel}}=0.11$ mm, making the object size about 3.75 cm. A cone beam geometry is used, where the source is placed 44 cm in front of the the center of the object, while the detector is placed 11 cm behind it. We use the National Institute for Standards and Technology (NIST) [63,70] attenuation spectra for each associated material to compute for each ray an approximation of the number of photons in energy bin $I(E_{i})$ hitting the detector. The computed quantity for each bin *i* with energy window $E_{i}$ and $1\leq i\leq N_{b}$ is given by the following:

(A1) $$\begin{array}{cc} I(E_{i}) & =\int_{E_{i_{\min}}}^{E_{i_{\max}}}I_{0}\left(E\right)e^{-\int_{\ell }\mu \left(x,E\right)dx}dE \end{array}$$

Here, $E_{i_{\min}}$ and $E_{i_{\max}}$ signify the energy range, $I_{0}\left(E\right)$ photon influx at energy *E*, *ℓ* is the ray trajectory and μ(x,E) is the attenuation at position *x* at energy *E*. This is approximated by inserting the assumption that μ(x,E) can be written as a linear combination of individual material attenuations:

$$\begin{array}{cc} \mu (x,E) & =\sum_{m\in M}\mu_{m}\left(E\right)\;\alpha_{m}\left(x\right)\\ \; & =\sum_{m\in M}\left(0.01{\bar{\mu }}_{m}\left(E\right)+0.99\mu_{\mathrm{polyethylene}}\left(E\right)\right)\alpha_{m}\left(x\right) \end{array}$$

where ${\bar{\mu }}_{m}\left(E\right)$ is the attenuation coefficient of material m∈M, with M being the set of involved materials, and $\alpha_{m}\left(x\right)$ the fraction of material *m* at position *x*. Inserting this into (A1) gives:

$$\begin{array}{cc} I(E_{i}) & =\int_{E_{i_{\min}}}^{E_{i_{\max}}}I_{0}\left(E\right)e^{-\int_{\ell }\sum_{m\in M}\mu_{m}\left(E\right)\;\alpha_{m}\left(x\right)dx}dE\\ \; & =\int_{E_{i_{\min}}}^{E_{i_{\max}}}I_{0}\left(E\right)e^{-\sum_{m\in M}\mu_{m}\left(E\right)\int_{\ell }\alpha_{m}\left(x\right)dx}dE \end{array}$$

The integral is numerically approximated using the midpoint rule and equally sized integration bins, which gives the following:

$$\begin{array}{cc} I(E_{i}) & \approx \sum_{j=1}^{N}I_{0}\left({\tilde{E}}_{j}\right)e^{-\sum_{m\in M}\mu_{m}\left({\tilde{E}}_{j}\right)\int_{\ell }\alpha_{m}\left(x\right)dx}\left(E_{i_{\max}}-E_{i_{\min}}\right) \end{array}$$

where *N* is the number of integration bins, and ${\tilde{E}}_{j}=E_{i_{\min}}+\frac{2(j-1)+1}{2n}\left(E_{i_{\min}}+E_{i_{\max}}\right)$ the average energy in the *j*-th integration bin. The number of integration bins is set to N=30 for this computation. The integral $\int_{\ell }\alpha_{m}\left(x\right)dx$ is computed using ASTRA.

The photon influx $I_{0}\left(E\right)$ is a product of the source spectrum ${\bar{I}}_{0}\left(E\right)$ at energy *E*, the exposure time *t* and the detector pixel size $s_{\mathrm{pixel}}$:

$$\begin{array}{cc} I_{0}\left(E\right) & =t{\bar{I}}_{0}\left(E\right)s_{\mathrm{pixel}}^{2} \end{array}$$

The exposure time is chosen to be t=0.5 s, and the source spectrum ${\bar{I}}_{0}$ is simulated as a radiology source spectrum for a thungsten source without filter at 70 kV, taken from Siemens Healhtineers [71]. The energy range used for this dataset is from $E_{1\min}=14$ kV to $E_{{N_{b}}_{\max}}=69$ kV, and the source spectrum including this range is given in Figure 8b. The final projection images in bin *i* are computed by dividing $I(E_{i})$ by the flatfield image $I_{\mathrm{flat}}\left(E_{i}\right)$ containing reference photon counts without objects

$$\begin{array}{cc} \frac{I(E_{i})}{I_{\mathrm{flat}}\left(E_{i}\right)} & =\frac{I(E_{i})}{\sum_{j=1}^{N}I_{0}\left({\tilde{E}}_{j}\right)\left(E_{i_{\max}}-E_{i_{\min}}\right)} \end{array}$$

## Appendix B. Robustness

Since all experiments in this work are not averaged over multiple runs due time computation time restrictions, we assess in this appendix the stability and robustness of a number of selected experiments. Included in this selection are the experiments where we witnessed the largest variation in the test results. We compute the average, standard deviation, minimum, maximum and median values of the average class accuracy over 8 different runs. The outcomes are given in Table A1. For each experiment, the standard deviation is at most 1.24, and for DRCNN methods this is 0.73. The difference between the minimum and maximum values is at most 3.51, and for DRMSD methods this is 1.38. From these results, we conclude that all the methods presented here are sufficiently stable, and these stability properties may be expected from the other experiments in this paper as well.

**Table A1:** Average, standard deviation, minimum, maximum and median of the average class accuracy for various network setups, computed over 8 runs.

Dataset	Data Type	Red. Type	Red. Chan.	Avg.	Std.	Min.	Max.	Median
X-ray	Noisy Many materials	DRMSD	2	99.30	0.0883	99.13	99.42	99.31
X-ray	Noisy Many materials	DRUNet	2	98.87	0.2937	98.36	99.29	98.97
X-ray	Noisy Many materials	LDA + MSD	2	94.09	0.7639	92.76	95.48	93.91
X-ray	Noisy Many materials	LDA + UNet	2	87.24	0.6757	86.10	88.21	87.36
Remote sensing	Noisy Overlapping	DRMSD	1	95.85	0.3642	95.28	96.34	95.86
Remote sensing	Noisy Overlapping	DRUNet	1	94.46	0.7250	93.27	95.58	94.65
Remote sensing	Noisy Overlapping	LDA + MSD	1	59.34	1.2328	56.27	60.60	59.78
Remote sensing	Noisy Overlapping	LDA + UNet	1	61.11	0.7674	60.03	62.48	61.05
